# Exploring the Lived Experiences of Medication for Opioid use Disorder Treatment: A Qualitative Study among a Crowdsourced Convenience Sample

**DOI:** 10.1007/s10597-024-01345-9

**Published:** 2024-09-05

**Authors:** Grant Victor, A. Kheibari, J. C. Strickland

**Affiliations:** 1 School of Social Work, Rutgers University, 120 Albany St, New Brunswick, NJ 08901 USA; 2 School of Social Work, Wayne State University, 5447 Woodward Ave, Detroit, MI 48202 USA; 3 Department of Psychiatry and Behavioral Sciences, Johns Hopkins University School of Medicine, 5510 Nathan Shock Drive, Baltimore, MD 21224 USA

**Keywords:** Opioid use disorder, Buprenorphine, Methadone, Treatment, Qualitative, Crowdsourcing

## Abstract

Given the effectiveness of medication for opioid use disorder (MOUD) and low engagement of treatment among people who use drugs (PWUD), it is important to better understand how to engage treatment clients with MOUD care. The current study aimed to achieve this goal by using qualitative methodology to characterize the MOUD treatment experiences. Participants (*N =* 52) were recruited for an online semi-structured interview. Qualitative analysis revealed varied treatment experiences, with the majority expressing irregular and intermittent MOUD treatment engagement. The therapeutic effects of MOUD in curbing withdrawal symptoms in conjunction with counseling services was frequently mentioned, as well as a preference for methadone maintenance treatment (MMT) to buprenorphine or naltrexone. Many participants described barriers to treatment and continuation of care, including failed drug screens for non-opioid drugs, perceived stigma, and physician-initiated discontinuation of treatment. The current study revealed that patients had favorable experiences with MOUD treatment, particularly when supplemented with counseling services.

## Introduction

The United States (US) is amid an overdose crisis as approximately 107,500 died of an overdose in 2023 (Centers for Disease Control and Prevention, 2024. In 2022, an estimated six million people aged 12 or older had an opioid use disorder (OUD) in the US, and approximately 1 in 250 people reported misusing fentanyl (Substance Abuse and Mental Health Services Administration, 2022). Despite the demonstrated effectiveness of MOUD (Connery, 2015; Connock et al. 2007; Fullerton et al. 2014; Ma et al. 2019; Mattick et al. 2003, 2009), access remains an issue for many patients with OUD.

There continues to be a gap in connecting individuals who use drugs and need care to medication for opioid use disorder (MOUD) treatment. In 2022, an estimated 3.7% of U.S. adults needed OUD treatment (Dowell et al. 2024), yet among this group, 55.2% received OUD treatment, and only about one-quarter (25.1%) received medications for OUD (Dowell et al. 2024). Moreover, prior research indicates that the use of MOUD within treatment systems is lower in states with higher opioid mortality, especially in areas with high rates of poverty and high rates of heroin and fentanyl availability (Huhn et al. 2020). Financial burden, stigma (including perceived stigma), regulatory barriers, and misconceptions about effectiveness of MOUD have been found to drive its limited use within treatment facilities (Sharma et al. 2017; Wakeman & Rich, 2018). In a study examining buprenorphine access and utilization among those with OUD, participants indicated that a lack of health insurance or other payment method, lack of access to free or reduced-price medication, and absence of access to a prescriber were barriers to receiving buprenorphine (Evans et al. 2019). Further, this study found that participants’ knowledge and perception of buprenorphine acceptability and accessibility had an impact on the likelihood of treatment utilization (Evans et al. 2019), indicating that increased education about MOUD effectiveness and access may be correlated with increased utilization.

Recent federal-level policy changes provided opportunities for increased access to MOUD. Following the onset of the COVID-19 pandemic, federal agencies (i.e., Drug Enforcement Administration [DEA] and Substance Abuse and Mental Health Services [SAMHSA]) implemented sweeping policy changes which eliminated the requirement that physicians needed a waiver to prescribe buprenorphine, lifted the requirement for an initial face-to-face consultation for prescribing buprenorphine and extended the permissible period for take-home methadone doses (Austin et al. 2023; Dowd et al. 2022), although some states have since rescinded these changes (Dowd et al. 2022). Of note, some states have already rescinded some of these policy changes that increased access to MOUD (Barsky et al. 2023).

### Current Study

Given the known effectiveness of MOUD and the relatively low engagement of treatment among patients with OUD, there is a need to better understand how to engage treatment clients with MOUD care on a long-term basis and to provide meaningfully positive treatment outcomes (Dillon et al. 2020; Yang et al. 2018). Pursuant to this goal is the need to give voice to the first-hand experiences of MOUD patients as they offer a critical perspective on the factors that may improve MOUD intake, retention, engagement, and satisfaction. The current study aimed to contribute to the current literature by using qualitative methodology to characterize the MOUD treatment experiences among a crowdsourced convenience sample of individuals with historical and current MOUD treatment engagement.

## Methods

### Screening and General Procedures

Participants were recruited with non-probabilistic convenience sampling methodology using Amazon’s Mechanical Turk (MTurk) from March 4, 2019 to May 1, 2019. This qualitative study used a phenomenological research design (Neubauer et al. 2019) to explore individualized experiences of MOUD treatment engagement. This research design approach was selected to facilitate an examination of the phenomenon of MOUD service utilization within participants’ broader social and structural contexts.

Amazon MTurk is an online crowdsourcing platform that provides researchers with a reliable method to recruit individuals for survey participation (Crump et al. 2013). Eligibility to view the study invitation on the MTurk platform was limited to individuals from the US who had completed 100 or more prior MTurk tasks with a 99% or greater approval rating on those tasks. Completion and approval criteria were used to enhance quality and attention and were used consistent with prior uses and recommendations on MTurk (Kaplan et al. 2017; Morean et al. 2017; Peer et al. 2014; Strickland & Stoops, 2019). Participants then completed a screening questionnaire that included questions about their substance use history and other health behaviors, like dietary and sleep habits (to further mask eligibility criteria). The inclusion criteria for this study were (1) age 18 or older, (2) lifetime non-medical prescription opioid use, and (3) transition to heroin and/or fentanyl use following non-medical prescription opioid use initiation (95% of participants reported heroin use). Qualifying participants were directed to full study questionnaire, which contained questions about a range of topics, including demographic information, substance use history, the impact of nonmedical prescription drug use on health, chronic pain, opioid overdose knowledge, opioid use history, and opioid use disorder treatment.

### Qualitative Questions: Opioid Use and Opioid Treatment History

Participants completed a series of semi-structured and open-ended questions about their opioid use history. These questions were devised to record beliefs and behaviors relevant to non-medical prescription opioid use, transitions to heroin or fentanyl use, and treatment encounters. The interview guide protocol for the current study included several broad domains, such as: “Please describe, in as much detail as possible, why you transitioned from using prescription opioid medication to using heroin and/or fentanyl”; “Please describe, in as much detail as possible, the process of obtaining heroin, fentanyl, or treatment medications. If appropriate, elaborate on how this process has changed over the years”; “If appropriate, please describe how you have managed the potential harms of your heroin and/or fentanyl use”; “If appropriate, please describe any drug-related contact you have had with health care setting(s) (Ex: emergency room visit, HIV screening test, etc.)”; “If appropriate, please describe your use of drug treatment services, such as counseling, Naloxone/Suboxone, syringe exchange programs, etc.” These interview questions were informed by the authors’ backgrounds in social work, behavioral pharmacology, and public health, as well as prior studies (Ciccarone, 2017; Cicero et al. 2018; Cicero & Ellis, 2017; Mars et al. 2014). Instructions stipulated that there were no length requirements, but to be as thorough as possible. Participants were also told that they did not have to answer a question if it did not apply (e.g., if they had no contact with the criminal justice system).

All participants were compensated $0.05 for completing the study screener and qualifying participants were compensated $5 for completing the full study. The University of Kentucky Institutional Review Board reviewed and approved all study procedures. See a detailed overview of the recruitment process and validity of this approach in (Strickland & Victor, 2020). The University of Kentucky Institutional Review Board reviewed and approved all study procedures. A description of participants’ demographic characteristics can be found in Table 1.

**Table 1:** Participant demographics and substance use

	Overall	No MAT (*N* = 21)	MAT (*N* = 39)
Age	34.2 (9.1)	34.9 (9.9)	32.8 (7.3)
Female	56.7%	59.0%	52.4%
White	81.4%	78.9%	85.7%
High School	57.6%	55.3%	61.9%
Income	3.4 (2.8)	3.0 (2.5)	4.2 (3.2)#
Housing			
Own a Home	26.7%	25.6%	28.6%
Rent a Home	48.3%	53.8%	38.1%
Live with Family/Friends	23.3%	20.5%	28.6%
No Permanent Residence	1.7%	0.0%	4.8%
Geographic Region			
Northeast	20.0%	12.8%	33.3%#
South	40.0%	51.3%	19.0%*
Midwest	26.7%	25.6%	28.6%
West	13.3%	10.3%	19.0%
Past Year Incarceration	15.0%	12.8%	19.0%
Past Month Heroin Use	45.0%	43.6%	47.6%
MOUD Use			
Ever Methadone	56.7%	51.3%	66.7%
Current Methadone	15.0	-	42.9%
Ever Buprenorphine	40.0%	28.2%	61.9%*
Current Buprenorphine	15.0	-	42.9%
Ever Naltrexone	35.0%	28.2%	47.6%
Current Naltrexone	11.7	-	33.3%

* *p* < .05

# *p* < .10

### Qualitative Analysis

The research team developed an established code construction protocol (Saldaña, 2015; Wicks, 2017). In the preliminary round of open coding, the research team read the transcripts of six participants, observing a preliminary list of codes that were concept-driven (emerging from extant literature) and data-driven (Saldaña, 2015; Wicks, 2017). Two authors (GV) and (AK) reviewed the data for patterns and discrepancies within each code that emerged most often within the transcripts and used nodes to define interrelationships between codes (Saldaña, 2015). The content coding process was refined at each reading interval such that each subsequent reading involved continual comparisons to construct existing codes with data in added transcripts by re-classifying or creating new code categories and definitions.

Once content coding reached a consensus, the research team further defined the codes with typical and atypical exemplars and identified the exclusion content per code. Two research team members tested inter-rater reliability using Cohen’s Kappa to calculate the degree of agreement for each code by adhering to an 80% agreement threshold. Qualitative data analyses were conducted using NVivo software (Lumivero, 2023). Using NVivo, the qualitative codes were checked for confirmatory or conflicting statements. Content saturation was reached after reading 52 transcripts from the 60 participants, so the final sample was *N* = 52. Participants’ narratives were presented verbatim, according to how they were recorded.

## Results

### MOUD Treatment Experiences

Although treatment experiences varied, most participants expressed irregular and intermittent SUD and MOUD treatment histories. The treatment services that were reported ranged from support groups, mandated treatment, residential rehabilitation, outpatient services, and MOUD treatment. A subgroup of the sample reported that they were currently receiving MOUD treatment, and fewer reported non-prescribed buprenorphine use as an ad-hoc treatment option – often acquired from peer groups (e.g., co-workers or friends).

The effectiveness of MOUD was often related to curbing withdrawal symptoms and cravings while facilitating outpatient treatment engagement both during MOUD uptake and after a tapering/discontinuation of pharmacology treatment. Notably, many participants revealed that their MOUD uptake reformed their conceptual understanding of the treatment capacity within addiction medicine, as described by a male from Indiana, *“I have been going for counselling and treatment over the last six months. The support system is great. It is good to know addiction can be treated. I was hopeless*,* but now I feel I can dream again.”* According to participants who had utilized MOUD uptake, the majority said that they preferred methadone maintenance treatment (MMT) to buprenorphine or naltrexone. However, a small number of participants mentioned that they preferred buprenorphine (Suboxone) to MMT due to greater receptor efficacy. A 52-year-old female from Pennsylvania described her MOUD treatment preference:

I saw my general practitioner and he started me on methadone. I felt like I was getting higher on that than I was on the small amount of drugs I was doing. So he put me on Suboxone. I started on 30 mgs day sublingual and eventually worked my way down. It took me two years to get off that, but it stopped the withdrawal and the cravings and absolutely saved my life.

The effectiveness of MOUD in conjunction with counseling services was evidenced by a female participant from New Hampshire, by saying, *“I’m prescribed suboxone through a wonderful doctor. I’m also seeing a mental health counselor and a mental health doctor for medication. I’ve never used opiates since being on suboxone.”* Although, MOUD treatment uptake was almost exclusively viewed as the most effective treatment option among participants who had experienced multiple recovery services. A 28-year-old female from Maryland described the contrasts in her treatment preferences:

I went to drug counseling for a few weeks in 2017. I felt like I wasn’t learning anything though, and it was boring so I stopped going. I was on suboxone from 2012 until 2017. It kept me away from pills the entire time and help me live a normal life. I started Subutex in September 2018 to get off of heroin, to stop withdraws, and to keep me from going back to it. I’m very happy with it so far. It’s been working to manage my cravings and again help me live a normal life.

### Pathways to MOUD Treatment

Participants described various pathways that led to their engagement with MOUD, including voluntary participation and court-mandated participation. In some instances, participants engaged in MOUD treatment only after attempting other programs/treatment options. Some participants indicated that their experiences with mandated treatment and support groups were their preferred treatment, such as a 36-year-old female from Alabama, who stated, “*I tried so many things. Methadone*,* suboxone*,* counselors*,* short term rehab. But what worked for me was a long term 6-month 12 step-based rehab facility*,* followed up with 12 step meetings. I was referred by the court. It saved my life.”* These participants often indicated that the structure and boundaries that were embedded in mandatory treatment programs benefitted their recovery. Further, some participants also noted that their compliance within mandatory treatment programs (e.g., following guidelines) validated and reinforced their commitment to recovery. Avoiding punishment was a motivating factor and perceived as a reward – similar to reward reinforcement in a contingency-based program but in the context of a mandated treatment model.

However, the consensus among participants was that mandated treatment was a difficult treatment pathway due to the lack of personal agency and punitive nature that undergirded their experiences. Participants consistently described disengagement and attrition in the context of mandated treatment programs – often to their legal detriment (i.e., fines and incarceration). A male participant from Massachusetts described his experiences with mandated treatment versus MMT, and how MMT compared to buprenorphine:

I have been through a variety of court-ordered rehabs, which never worked. Since everyone in the program was there because they were ordered by the judge, nobody actually wanted to get sober and continued to find ingenius (sic.) ways to be able to get high. These were an awful influence on me, even when I really wanted to get clean. Now that I am on methadone, it helps a lot to keep me from regular use, since it curbs the physical withdrawals, which are near impossible to deal with cold turkey. The clinic also requires me to attend individual and group counseling, which I find to be a waste of time for me personally. All it is, are the same common-sense topics being discussed repeatedly, so I do not feel like I receive any new benefit from it. I tried to take suboxone before but it did very little for my withdrawal symptoms.

Other participants described that their uptake of MOUD was in-part facilitated by external pressures such as familial and intimate relationships as well as legal pressure that influenced their MOUD uptake. A 32-year-old married male participant from Pennsylvania described his experience as the following:

I went to rehab once when I was arrested and the officer told my mom he wouldn’t throw me in jail if she promised to get me in rehab. My mom did get me in a 30-day program but I wasn’t ready and only stayed sober for 30 days after finishing program. I ended up going to the methadone clinic and was on the methadone clinic for three years. It was very helpful because I was getting the counseling and tools I needed while being medicated. It helped me build a plan while I was still being medicated so it made it so much easier to stay away once the time came to get off of the methadone. I was also going to individual and group therapy at another drug and alcohol program and would go three times a week to that program while going to the methadone program daily.

Another male participant, a 53-year-old from Iowa, described how recovery assistance from his intimate partner aided in his MOUD uptake:

Almost right after that my girlfriend starting stating very plainly that I needed help and she would help me get it. I found an outpatient service here that would get me on methadone and provide counseling also. At first it was many days a week and I had meetings with other addicts and a lot of things to keep me busy at home. As I did better i did not have to go everyday. I went down to 3 meetings a week, then 2… I am now only doing 1 a month and one meeting with my primary counselor. I also see the doctor there when needed. a special doctor is needed to provide methadone and he helps me to keep an eye on hep c stuff as well.

### Treatment Barriers

Another theme that emerged regarding MOUD uptake were various barriers to treatment, such as stigma, high costs, strict treatment program requirements, and inconsistencies in the services provided by medical professionals. Several participants said that their continuation of MOUD uptake was impeded by failed drug screens for non-opioid drugs (e.g., Tetrahydrocannabinol [THC]) and out-of-pocket costs. In addition, some participants described physicians’ “randomly” reducing or discontinuing their MOUD treatment – a practice that was described as “*inadvertently making things worse*.” A belief that treatment providers have negative attitudes towards people who use drugs (i.e., perceived stigma) was also noted as a barrier to accessing treatment. A 35-year-old female from Massachusetts, who reported interruptions in MMT uptake, claimed that she felt stigmatized by medical providers after being wrongfully accused of failing to meet drug test requirements:

At first I went to detox but since I was pregnant, they couldn’t detox me. They kept me on maintenence (sic.) and I had the decision of whether to stay on it or not after I had my baby. I chose to stay on it, and I’m not gonna lie, if I were’t (sic.) on it right now still, I would be using. However, it comes at a cost. I have tested positive for THC twice since I’ve been there, and I don’t smoke at all. I haven’t smoked pot since I was 15. I had 13 take home bottles (maximum) for 12 years and just recently lost them all 2 months ago because of this. It must have been someone else’s sample because I’m not even around it. I tried to fight it, but according to them, we’re all lying cheating loser junkies so nothing we say is taken into consideration. I go to all my groups, counseling, medical appointments, etc… I’ve been clean for 12.5 consecutive years. Year 6 I tested positive for THC, lost my bottles for 30 days but got them all back afterwards. This time, the second time I tested positive for something I didn’t smoke, they took my bottles again but this time they didn’t give them back.

While stigma was noted as a general concern when interacting with community members and in other medical settings (e.g., primary care), a greater number of participants described feeling stigmatized in opioid treatment programs (OTP) specifically. There were several responses that noted how attending OTPs reinforced stigmatizing attitudes toward them, such as being labeled an *“addict”* or *“untrustworthy.”* Participants also reported feeling stigmatized when filling their MOUD prescriptions as they believed the pharmacy staff made negative judgments about their character and intentions for seeking medical care. It is important to note that these stigmatizing experiences were based on how the participants made meaning of their interactions with others; hence, the emphasis on perceived stigma.

Participants’ descriptions of MOUD discontinuation were also saturated with cynicism about alternative treatment options and the surplus of resources that were required to maintain MOUD care. For instance, participants described moderate to poor experiences in counseling and support groups; yet many were dismayed by the barriers to quality counseling services in lieu of MOUD (e.g., high costs) and the perceived arbitrariness of primary care providers practice decisions to discontinue MOUD treatment. A male from Oregon described his experiences by saying:

At 16 I went to an inpatient rehab which was a joke and actually tuaght (sic.) me about different ways to use or get drugs. Counseling is a joke unless you have a lot of money the Drs may have good intentions but sometimes inadvertently make things worse by randomly deciding to stop giving suboxone or drastically reducing the amount.

## Discussion

The findings from this qualitative analysis revealed that encounters with MOUD treatment were primarily a positive experience MOUD but barriers to these treatment modalities were common as numerous participants expressed that logistics (e.g., out-of-pocket costs), policy (e.g., “first fail” abstinence-based treatment, dosage tapering/discontinuation), and perceived stigma adversely impacted their utilization of MOUD. Our findings suggest there was minimal support for mandated treatment – although some found this structure beneficial – especially when it circumvented their access to MOUD. Consistent with prior literature, participants who reported past or present MOUD histories were generally supportive of these treatment modalities (Cioe et al. 2020; Fox et al. 2015; Hewell et al. 2017). However, despite the robust evidence that has demonstrated the effectiveness of MOUD in reducing drug-related mortality (Connery, 2015; Connock et al. 2007; Fullerton et al. 2014; Larochelle et al. 2018; Ma et al. 2019; Morgan et al. 2019; Wakeman et al. 2020), barriers to care were persistent for the current study sample, and the narratives expressed were congruent with previous studies (Duncan et al. 2015; Rawson et al. 2019). Finally, there was little indication that MOUD was used for any psychoactive or “high” effects.

Although data collection predated the COVID-19 federal-level removal of regulatory restrictions to MOUD (i.e., buprenorphine and methadone), there is evidence from the current study that these changes would have been received favorably by our sample. Participants noted poor experiences in MMT settings and increased take-home doses could alleviate the frequency of opioid treatment centers and improve feelings of trust and autonomy (Hoffman et al. 2022). Stigma and negative attitudes toward MOUD treatment were reported with particular relevance to OTPs, as was the case in a previous study (Rawson et al. 2019). Addressing negative attitudes on behalf of providers or patients is essential because they may suppress MOUD uptake even when other barriers (e.g., cost) are mitigated (Molfenter et al. 2015). It is also important to note that Medicare Part D claims data suggest that buprenorphine providers who initiate prescribing buprenorphine continue to do so fairly consistently, which has implications for improving access and creating a comfortable environment for individuals seeking treatment (Huhn et al., 2020).

Our findings may have implications for individualized MOUD treatment planning. Overall, the participants’ treatment goals were primarily based on a desire to avoid withdrawal symptoms, to reduce problematic opioid use, and to stabilize their lives to facilitate a successful recovery. Although there is a lack of evidence supporting MOUD tapering compared to ongoing maintenance treatment of MOUD (Fiellin et al. 2014; Nielsen et al. 2013), many participants described MOUD tapering during a period of treatment, but it is unclear how tapering may have been formulated as a treatment goal between patient and provider. Future research might explore the congruence between patients’ treatment expectations and providers’ treatment planning. Several participants also noted that their MOUD preference (i.e., buprenorphine or MMT) was discovered following a period of trial-and-error. This may suggest the need for MOUD patient decision guides to increase knowledge of treatment options and to facilitate shared decision-making between patient and provider (Mooney et al. 2020).

Participants’ preferences and opinions of MOUD with adjunctive treatment services (e.g., psychosocial interventions) were varied. Some participants lauded the support and structure they gained from counseling services, while others described it as “a waste of time.” Previous research has illustrated that there is little evidence supporting the need for psychosocial interventions with MOUD treatment, although prior research has found that individuals with OUD may prefer substance use disorder treatment. For example, Andraka-Christou et al. (2021) found that “ideal” treatment centers have a host of treatment options, including MOUD and peer support, multilevel integration with other care levels (e.g., residential), and adjunctive supports (e.g., housing and employment). These themes emerged in the current study, in that participants had varied experiences of MOUD with adjunctive services, but there was a desire to have treatment options alongside MOUD. However, there is ample evidence that suggests MOUD care alone improves treatment retention (Askari et al. 2020; Hadland et al. 2018; Manhapra et al. 2017; Mattick et al. 2003, 2009). However, there is ample evidence that suggests MOUD care alone improves treatment retention (Askari et al. 2020; Hadland et al. 2018; Manhapra et al. 2017; Mattick et al. 2003, 2009).

Numerous participants described gaps and structural barriers to maintenance treatment; such as, stigma, high costs, and physician discretion, which reinforces the notion that retention is multifaceted (Krawczyk et al. 2021; Victor et al. 2021). Our results underscore the need for individualized care when considering adjunctive psychosocial services so that they optimize the patient’s needs, and future research should consider randomized-controlled trials to better understand which, if any, psychosocial interventions may be most effective as adjunctive treatment options (Bassuk et al. 2016; Ray et al. 2021; Rice et al. 2020). Therefore, clinicians and providers may consider a psychosocial intervention with their patient as our data support, but the primary treatment objective in most cases should be to initiate or continue MOUD – the underutilized gold-standard of care for patients with OUD (Beetham et al. 2020; Wakeman et al. 2020).

### Limitations

This study had several noteworthy limitations. Although this non-probabilistic convenience sample included participants from across the United States, the sample size was relatively small and restricted to those with access to a computer; thus, limiting the generalizability of the results. In addition, the findings presented in the current study may be biased given our sampling procedure, such that the resulting sample may not have captured a broad variety of perspectives among individuals who engaged in MOUD treatment. More broadly, the use of crowdsourcing resources may present sampling bias and lack of control over the testing environment (see discussion of crowdsourcing strengths and limitations in (Chandler & Shapiro, 2016; Strickland & Stoops, 2019). Importantly, prior work has found that MTurk samples do not engage in higher rates of dishonest or disingenuous behavior (e.g., responding in socially acceptable ways or without paying attention) than other convenience samples like college or community samples (Necka et al., 2016). The nature of the open-ended survey did not allow for real-time follow-up questions for added breadth, clarity and context of personal narratives which may limit the scope of participants’ reports.

## Conclusions

In support of expanded MOUD access, participants in our sample indicated that MOUD care was effective and favorable and there was little indication that misuse was the primary motivation for MOUD consumption. The demand and treatment effect of MOUD was high – especially for methadone and buprenorphine. Federal-level policy changes to MOUD access since the onset of COVID-19 were reinforced by our results – particularly regarding the less- stringent methadone take-home policies – although data collection for the current study pre-dated these changes. Future discussions aimed at retaining or expanding COVID-19 MOUD policy changes may have a greater impact if the voices of patients are elevated in state- and local-level discussions. Since these conclusions can only be generalized to the present sample due to the limitations of the sampling procedures, future research should aim to replicate these findings using a more representative sample.
